# Exploring cultural factors contributing maternal mortality among pregnant women: an ethnographic study in the Banjarnegara community, Central Java, Indonesia

**DOI:** 10.3389/fgwh.2025.1677072

**Published:** 2025-10-28

**Authors:** Dewie Sulistyorini, Mayumi Kako, K. A. T. M. Ehsanul Huq, Zahroh Shaluhiyah, Michiko Moriyama

**Affiliations:** 1 Graduate School of Biomedical and Health Sciences, Hiroshima University, Hiroshima, Japan; 2 Diploma III, Midwifery Study Program, Banjarnegara Polytechnic, Banjarnegara, Central Java, Indonesia; 3 Department of Health Promotion, Faculty of Public Health, Diponegoro University, Semarang, Central Java, Indonesia

**Keywords:** cultural practice, pregnant women, community, health worker, shaman

## Abstract

**Background:**

Cultural beliefs and power relationships existing in family daily practices significantly influence the health behaviors and outcomes of pregnant women. The role of family hierarchy and shamanic practices in shaping maternal health behaviors. In rural Indonesian communities, limited access to education and health literacy among women and their families hinders informed decision-making, increasing the risk of maternal and neonatal complications and death. This study approach, informed by the Foucauldian view of power relationships within family dynamics, aimed to explore the cultural norms and daily practices that contribute to adverse pregnancy outcomes.

**Method:**

A qualitative ethnographic study was conducted in Banjarnegara, Indonesia. Seventy participants—including pregnant women, their husbands, health cadres, and midwives—were recruited from three Public Health Centers (PHCs). Data collection involved 12 focus group discussions (FGDs), field observations, and in-depth semi-structured interviews. Data was analyzed by utilizing discourse analysis that highlighting communication and interactions of pregnant women with family members and health cadres who are assigend to support the women. This study was reported according to the COnsolidated criteria for REporting Qualitative Research (COREQ).

**Results:**

Thematic using discourse analysis revealed three primary themes: 1) daily activities of pregnant women, 2) family hierarchy and power dynamics, and 3) cultural practices involving shamans during pregnancy. The most frequently coded subthemes were cultural food practices (48.6%); activity-related practices (37.1%); family dominance (28.6%); shamanic practices (15.7%); practices related to rest and sleep (14.3%); and lack of reproductive control (14.3%).

**Conclusion:**

Pregnant women were often subject to culturally driven food taboos and restrictive physical routines, heavily influenced by family hierarchies and power relationships existing in their families—particularly mothers-in-law and husbands. These influences extended to decisions about reproductive health and prenatal care, sometimes leading to unplanned pregnancies and unsafe practices. The role of shamans, while culturally significant, poses risks when traditional methods conflict with scientific standards of care. Strengthening communication between healthcare providers and families, promoting culturally sensitive education, and empowering women through targeted interventions are essential to improving maternal and neonatal outcomes in these communities.

## 1 Introduction

In recent years, Indonesia has experienced a decline in its overall maternal mortality ratio (MMR). In 2023, Banjarnegara Regency reported an MMR of 125.6 per 100,000 live births. Of the maternal deaths recorded, 33% occurred during pregnancy, 13% during labor, and 53% during the postpartum period, the period beginning immediately after childbirth, specifically following the delivery of the placenta. Postpartum hemorrhage remains a major complication during this phase, influenced by maternal, obstetric, and healthcare-related factors. Hemorrhage, often associated with anemia, is a leading cause of maternal mortality (1). To prevent anemia, antenatal supplementation is recommended. However, the prevalence of anemia among pregnant women in Indonesia remains high, affecting approximately 37% (2). A balanced diet during pregnancy is essential in mitigating this risk.

Maintaining a healthy pregnancy is critical for ensuring favorable maternal and neonatal outcomes. Cultural beliefs and practices, shaped through interpersonal communication within families and communities, play a significant role in influencing maternal health behaviors. These cultural norms strongly affect dietary choices during the perinatal period and may be modified by factors such as social dynamics, health literacy, medical conditions, and family finances (3). However, the extent to which cultural values influence pregnant women's health behaviors has not been fully explored. The role of healthcare providers—including midwives and community health cadres—is crucial in addressing culturally driven beliefs and behaviors. Educating family members, particularly husbands and mothers-in-law, who often act as co-decision-makers in maternal care, is essential for supporting maternal health (4). There are lack of studies exploring maternal health behaviors from a cultural perspective, especially in rural Indonesia. There is also a paucity of studies analyzing how pregnant women and their family relationships are impacted by power within the smallest unit of community. Therefore, this study aimed to explore the cultural beliefs and practices affecting pregnant women in Banjarnegara, Indonesia.

## 2 Methods

### 2.1 Study design

This study employed an ethnographic qualitative research design conducted between February and December 2023. The design was also informed by how Michel Foucault's understand governmentality and power at all levels of society (5). This approach allowed for a deeply contextualized understanding of the participants of daily life who practiced under a cultural environment. Foucault's perspective on power relationships in society at all levels, in this study, it focuses on pregnant women and their families which consists of the micro level of community. Discourse analysis including health cadres, who are assigned to support pregnant women as volunteers, will be able to contextualize the relationship and its dynamics embedded in power relationships (6). The Principal investigator (PI) was also aware about the cultural perspectives of the pregnant women and their family practice from an emic (as a resident from the study area: inside) and an etic perspective (currently staying outside of that area). Data collection involved focus group discussions (FGDs) and field observations, which were well-suited to exploring how cultural beliefs and practices influence pregnancy-related behaviors. There is a total of 35 public health centers (PHCs) in Banjarnegara, 10 in the urban and 25 in the rural areas, where 13 of those are dealing with basic emergency obstetric neonatal care. These PHCs serve maternal and child health including antenatal care, delivery, family planning and immunization among the community people. We selected one public health center (PHC) from an urban area and two from rural areas through convenience sampling to obtain a representative sample of different community settings. PI knows the situation and facilities of PHCs and uses these three centers for field practice of the midwifery students. This study was reported according to the COnsolidated criteria for REporting Qualitative research (COREQ) (7).

### 2.2 Study procedures

The PI (First author) personally visited the heads of the selected Primary Health Centers (PHCs) to obtain formal approval for conducting this study. At each PHC, the PI requested the coordinator midwife, who served as the site manager, to compile a list of potential participants, including midwives, health cadres, pregnant women, and their husbands and mothers-in-law. The PI then contacted the identified individuals and obtained written informed consent for their participation in the focus group discussions (FGDs).

At each PHC, four participant groups were conveniently selected: (1) pregnant women who lived in the study site at the time of recruitment, aged 19 years old and above, pregnant women at their any pregnancy, gestational age 16–40 weeks, had husbands and mothers-in-law, and had regular access to their designated public health center at one of the study sites, (2) husbands who lived together and mothers-in-law who lived together or nearby of the pregnant women, (3) health cadres who had been working at the study site for at least 5 years, and (4) midwives who had been working at least 5 years in the public health centers at the study site. All groups participated in the FGDs, while only the pregnant women were involved in field observations. The FGDs and field observations were facilitated by the PI—a female doctoral student, mother of two children, and has experience in qualitative research. She has served as a midwifery lecturer at a local college in Banjarnegara, Indonesia, for 16 years and is well-acquainted with the midwives and health cadres in the PHCs due to her residence and clinical practice in the community. The second author, who holds a master's degree in anthropology and lectures at the same college, has 16 years of qualitative research experience in health sciences.

### 2.3 Focus group discussions (FGDs)

A total of 12 FGDs were conducted across three PHCs. Each FGD involved four groups, with six participants per group, resulting in a total of 70 participants (two mothers-in-law declined participation). We tried to understand the insights, perceptions, beliefs and practices in the perspectives of community people (pregnant women, their husbands and mothers-in-law) and healthcare providers (midwives and health cadres) from three different PHCs. For that, we thought these participants' responses would be sufficient to answer our research question. Each session lasted approximately 1.5 to 2 h. Four FGDs were held at the PHCs, and eight were conducted in village meeting halls, as some PHCs were located far from the participants' communities. Private rooms were arranged at the PHCs, and confidentiality was ensured at the village halls to facilitate open and comfortable discussions. The FGDs were observed by an anthropologist who documented nonverbal expressions, gestures, and body language. Additionally, a trained professional video-recorded each session with participants' consent. The PI introduced herself, explained the study objectives, and facilitated the sessions using a structured interview guide (Table 1). Repetitive interviews were not conducted, as data saturation was achieved—no new information emerged beyond the existing data. The interview guide was developed based on the study objectives and validated by three experienced qualitative researchers: a university midwifery lecturer, a PHC head midwife, and a midwife from the Regency Health Department.

**Table 1:** Interview guides for FGDs.

No	Group discussion
1	For pregnant women
	Please explain your habits of eating, what kind of food? How many times do you eat?
	Please explain the habits of rest and sleep, if napping how many hours? How many hours do you sleep at night?
	Please explain the habits of cultural activities during pregnancy?
	Please explain the habits of work/ housework
	Please explain the habits of traveling anywhere during pregnancy. What kind of transportation do you use?
	Please explain the source of information about pregnancy
2	Husband and mother-in-law
	How did you feel when you found your wife was pregnant?
	What activities a pregnant woman does during their pregnancy?
	What activities are prohibited for pregnant women during pregnancy?
	What is the role of a shaman for pregnant women?
3	Health cadre
	Motivation to become a health cadre?
	Please explain the traditions, myths, or beliefs that pregnant woman should know that those are harmful for them such as eating habits
	Please explain the traditions, myths, or beliefs that pregnant woman should know that those are harmful for them such as social activities
	Please explain the traditions, myths, or beliefs that pregnant woman should know that those are harmful for them such as power of the family members for decision-making related to pregnancy
	Please explain the traditions, myths, or beliefs that pregnant woman should know that those are harmful for them such as traveling
	Please explain the traditions, myths, or beliefs that pregnant woman should know that those are harmful for them such as rest and sleep
4	Midwives
	Please explain the traditions, myths, or beliefs that pregnant woman should know that those are harmful for them such as eating habits
	Please explain the traditions, myths, or beliefs that pregnant woman should know that those are harmful for them such as social activities
	Please explain the traditions, myths, or beliefs that pregnant woman should know that those are harmful for them such as family hierarchy and power control
	Please explain the traditions, myths, or beliefs that pregnant woman should know that those are harmful for them such as traveling
	Please explain the traditions, myths, or beliefs that pregnant woman should know that those are harmful for them such as rest and sleep

### 2.4 Field observation

After FDGs, we conducted field observations to understand and explore the hidden, unspoken and unconscious behaviour in their own environment (home). This observation provided the researchers to understand participants' cultural daily living and their interactions with families. Three pregnant women were selected from each of the three PHCs (totaling nine participants). Initially, one pregnant woman from each PHC who had joined an FGD was asked to invite two additional pregnant women from her community to participate in the observation (snowball sampling). We chose two new pregnant women from each PHC (total 6), along with one (total 3) who participated in the FGDs, to increase the number of participants and to gather more new information. These three original FGD participants also participated in field observations. Following the FGDs, the research team visited the participating nine pregnant women in their homes, where each observation lasted 1 to 2 h. During these visits, the PI conducted unstructured interviews by collecting data through observation and recording field notes (8). to gain insights into behavior and activities in their environment. PI asked the women to describe their typical 24 h routines, including dietary habits, daily activities, rest and sleep patterns, decision-making processes regarding maternal health, and traditional practices performed by local healers during pregnancy. We planned to continue conducting the observations until we reached the saturation point. For that, we did ongoing analysis during the data collection process. After completing all nine observations, we felt that we had achieved the study objectives, and no new information was gathered. We thought we had sufficient samples to reach the saturation point.

### 2.5 Data analysis

**Figure 1:**
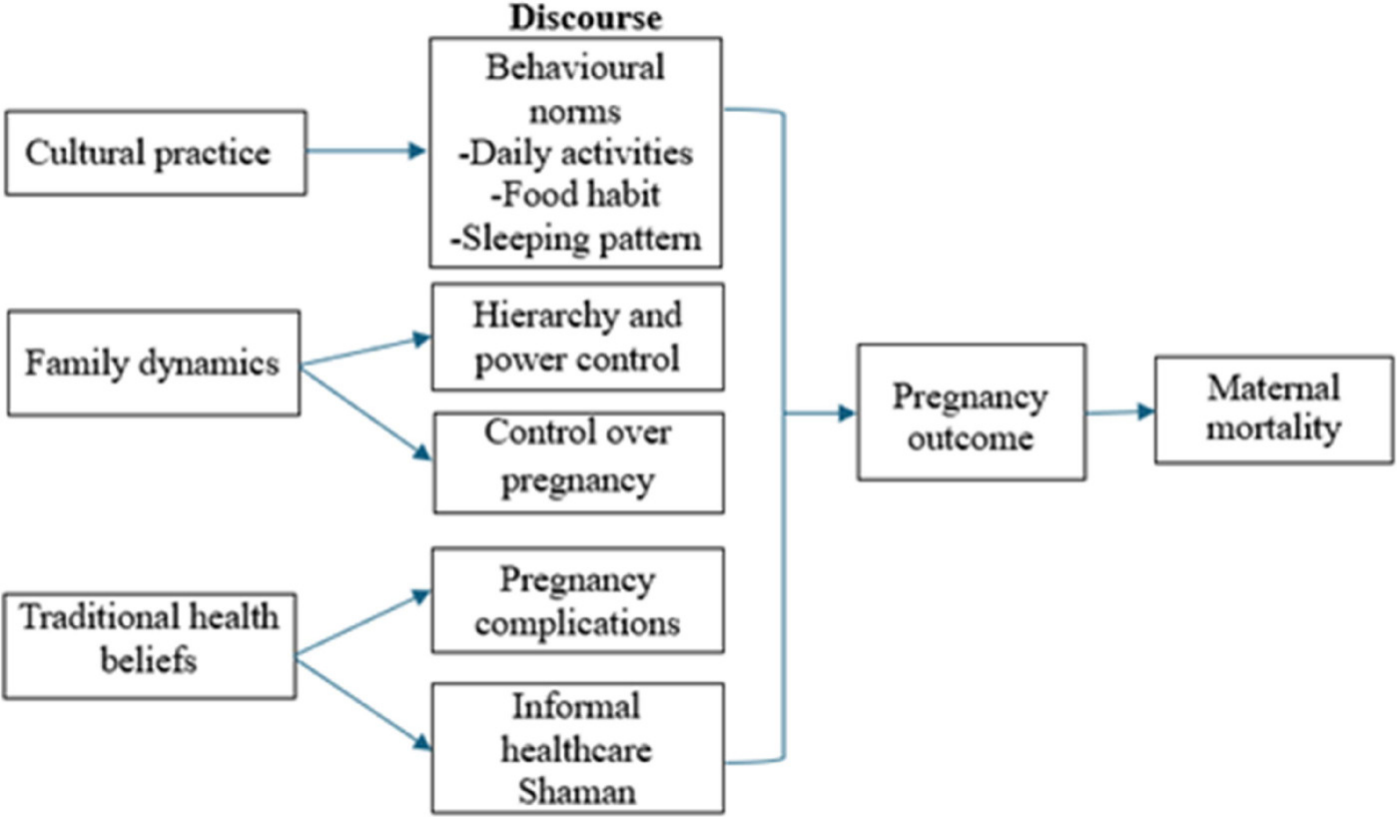
Theoretical framework of study concept.

All FGDs were video-recorded and transcribed verbatim into Indonesian and the local Javanese dialect. Field observations with in-depth interviews were documented by field notes. Data were managed strictly to ensure privacy, anonymity and confidentiality. Only PI and the research team had access to the data, and all study documents were kept under lock at respective sites. Data was entered using individual codes of the participants; no identifying information was documented. Thematic discourse analysis was performed using Dedoose, a cloud-based application (CA: Socio-cultural Research Consultants, LLC, https://www.dedoose.com). We used this tool as it analyzes the data in a more useful way to visualize data. The number of times each code was applied to each text, and the color coding helped us quickly spot the most frequently used codes. This analysis was completed with six phases (9, 10): In Phase 1, PI and research team familiarized themselves with data by reading and reviewing all transcripts carefully and repeatedly, and combined into one format. These transcripts were subsequently translated into English. Accuracy checks were conducted by an independent research team (four members), and corrections were made as necessary. A codebook was established manually after discussing it with the research team. Then upload the data via the “import data” in Dedoose. In Phase 2, PI and team created the codes by “adding root code”. In Phase 3, generated preliminary themes, reviewed the data coded. Phase 4, developing and reviewing themes, organizing the code book to develop initial themes. Phase 5, PI and team refined and defined themes, Phase 6, final write-up by exporting data and presenting findings used the code application tool. Predefined theoretical frameworks (Figure 1) with Foucault perspectives on power relationship within pregnant women and families, and how health cadres, as outside of family carers understand women and their family practice were used as discourse analysis. Themes and sub-themes were grouped according to key categories, and representative verbatim quotes were included to support findings and enhance transferability to similar contexts.

### 2.6 Trustworthiness

Trustworthiness including credibility, transferability, dependability, and confirmability were ensured in every step throughout the study (11). Both FGDs and field observations were conducted by experienced researchers, a midwife and an anthropologist both who specialize in maternal health. Most of the research team members had prolonged engagement with the participants and were familiar with community's cultural beliefs, rituals, and customs as they were residing there. It gave them confidence to explore the real scenario of cultural factors related to unhealthy beliefs and practice during pregnancy. We used FGDs, observation and in-depth interviews for triangulation to increase validity, reliability and credibility. The validation was conducted by a supervisor (last author) who holds a doctoral degree and possesses extensive experience in qualitative research, overseeing all study activities throughout the research process. Study findings were reviewed by co-authors with expertise in qualitative health research and midwifery education. We described study settings, context, sampling methods, data collection procedures and analysis that were appropriate to the research question. These enhance study transferability of our study in other geographical and cultural backgrounds. Dependability was ensured by proper documentation, maintaining consistency and stability of our research process throughout the study period. We assured confirmability relied on participants responses rather than researcher's understanding on data collection procedures and interpretation. Following data collection, the research team engaged in iterative discussions until consensus on findings was reached. PI was also aware that as a researcher who is from the area and this insider (emic) perspective may have influenced data collection and analysis. To avoid and minimize bias assumed by PI, a supervisor and independent team made sure that analysis and discussion was justified and well informed by the theoretical perspectives set out for this study.

## 3 Results

In total, 76 individuals participated in the study: 70 in the FGDs and 9 in the field (home-visit) observations with in-depth interviews (including 6 new participants and 3 who also participated in FGDs). Among all the participants, the mean ages were pregnant women (*n* = 18), their husbands (*n* = 9), mothers-in-law (*n* = 7), midwives (*n* = 18) and health cadres (18) were 27.7 years, 30.7 years, 58.1 years, 37.4 years and 38.3 years, respectively. More than half (55.6%) of the pregnant women completed junior high schooling, followed by senior high schooling (27.8%) and diploma and above (16.7%). Most of the pregnant women were housewives (94.4%), unemployed. The mean gestational age was 24.9 weeks and 33.3% had no children (no pregnancy experience) (Table 2). Data collection ceased once the research team determined that no new information was emergingDedoose analysis revealed six predominant cultural factors that could contribute to high maternal mortality among pregnant women (Table 3). The most frequent coded themes included harmful cultural food practices (48.6%), activity-related cultural practices (37.1%), family dominance (28.6%), reliance on shamans (15.7%), restrictions related to rest and sleep (14.3%), and lack of reproductive autonomy (14.3%), wherein women could not control the number of pregnancies due to limited empowerment. Data are presented with the following three themes:

**Table 2:** Participant characteristics.

Variables	Pregnant women (18)	Husbands (9)	Mothers-in-law (7)	Midwives (18)	Health cadres (18)
Age (mean ± SD)	(27.7 ± 5.7)	(30.7 ± 7.2)	(58.1 ± 7.2)	(37.4 ± 6.1)	(38.3 ± 8.3)
Education level					
No education	0	0	0	0	0
Elementary school (6 years of schooling)	0	0	5	0	2
Junior High School (9 years of schooling)	10	5	2	0	3
Senior High School (12 years of schooling)	5	2	0	0	12
Diploma and above (15 years and above)	3	2	0	18	1
Profession				–	–
Housewife	17	–	3	–	8
Employee (office)	1	3	0	–	0
Teacher	0	1	0	–	0
Farmer	0	4	3	–	8
Worker	0	1	1	–	0
Village officials	0		0	–	2
Work experience		–	–		
<10 years	–	–	–	2	
≥10 years	–	–	–	16	
Religion (Muslim)	All	All	All	All	All
Gestational age (weeks, mean ± SD)	(24.9 ± 7.6)	–	–	–	–
Number of children			–		
0	6	–	–	–	–
1	9	–	–	–	–
2	1	–	–	–	–
3	2	–	–	–	–

**Table 3:** Codes in the analyzed workspace.

Codes	FGD												
	Hus band Rakit	Hus band Peja waran	Hus band Banjar negara	Pregnant woman Rakit	Pregnant woman Pejawaran	Pregnant woman Banjar negara	Midwife Rakit	Midwife Pejawaran	Midwife Banjar negara	Health cadre Rakit	Health cadre Pejawaran	Health cadre Banjar negara	Total
Activities of health cadre										1			1
Activities of pregnant women				3		2							5
Cultural practice of the solution on miscarriage		2											2
Cultural practice of activities	2	3	3		6	4				7	1		26
Cultural practice of food	3	2	2	3	6	3		1		4	6	4	34
Cultural practice of rest and sleep	1		1	1	1		1	1		1	2	1	10
Family dominant	1	6		5	3				2		3		20
Family worries	3												3
Midwife advice		1	1				1		1	1			5
Midwife under standing about pregnant women cultural practice							1						1
Miss under standing about miscarriage		1											1
Cultural practice of shaman		4					1		4		1	1	11
Sources of information				3		4							7
Uncontrollable pregnancy	2				5	1	1	1					10
Cultural of day							2						2
Total	12	19	7	15	21	14	7	3	7	14	13	6	

Utilizing coding, PI developed three themes: (1) daily activities of pregnant women; (2) family hierarchy and power control over pregnant women and (3) cultural practice of shaman during pregnancy, with 6 codes (Table 4). To identify the statement of individual participants we used acronyms: B, banjarnegara; R, rakit; P, pejawaran; PW, pregnant women; H, husband; MIL, mother-in- law; HC, health cadre; M, Midwife.

**Table 4:** Themes and codes.

Themes	Codes
Daily activities of pregnant women	Cultural practices of food
	Cultural practices in activities
	Cultural practice of rest and sleep
Family hierarchy and power control to pregnant women	Family dominant
	Women cannot control their pregnancy
Cultural practice of shaman during pregnancy	The practice of shamanism

### 3.1 Theme 1: daily activities of pregnant women

Pregnant women and their families—particularly husbands and mothers-in-law—commonly adhered to food-related myths. Certain foods were labeled as either taboo or recommended, with mothers-in-law often instructing pregnant women on what to eat, when to eat, and in what quantities.

“When I was first pregnant, I was not allowed to eat shrimp, she said that if I gave birth, it would not go forward” (PWB5)

Health care personnel confirmed these beliefs:

“Eating eggs is also not allowed, mothers-in-law say the baby will have itchy skin”. (HCP5)

Midwives observed cases where pregnant women either were not permitted or were unwilling to eat protein-rich foods, leading to anemia:

“During pregnancy, they can’t eat fishy things like eggs or meat. Even though these foods can prevent anemia. It turned out when I asked [this], the pregnant woman was not allowed to eat fishy things” (MR5)

Further, pregnant women were sometimes forbidden from consuming fruits and vegetables due to beliefs that these foods would negatively affect childbirth. In many cases, restrictions were followed without clear justification:

“I am not allowed to eat pineapple like that, mother-in-law said, because it can cause a miscarriage”. (PWP4)

Cultural beliefs were held not only by families but also by some health cadres providing care to pregnant women.

“Pregnant woman can't eat melinjo leaves; during labour- the contractions will be slow”. (HCR6)

“Melinjo leaves, can cause bleeding when giving birth” (HCR3)

Although some food restrictions—such as avoiding high-fat foods and excess seasoning—may align with health advice, cultural reasoning often lacked a scientific basis:

“You can't eat too much fried food such as fried rice, the placenta can be sticky” (HCB5)

Certain cultural practices also involved protective rituals to safeguard the unborn child from supernatural harm. For example, pregnant women were instructed to carry specific items when going out at night:

“The myth is that when you go out at night, you are told to let your hair down, then you are told to bring scissors and a nail cutter (mother-in-law informed to pregnant woman)” (PWR6)

The health cadre also shared some common cultural practices as follows:

“When I was pregnant, I came home from my in-laws’ house, and she told me to bring scissors and put them in my shirt”. (HCR3)

Another widespread belief concerned daytime sleeping. Families believed that napping during the day could negatively impact both the mother's and baby's health. Pregnant women were not allowed to take a nap; the family believed that sleeping during the day was harmful for the pregnant woman and the baby's health:

“My husband and his parents said that I can’t take a nap” (PWP1)

Furthermore, another husband and mother-in-law did not allow the pregnant woman to take a nap too often because it could cause harmful condition to baby's health status:

“If you take a nap, don’t take it too often; if it's too frequent, it's said to reduce the baby's health” (HB2)

Health cadres also reported hearing such beliefs from older generations:

“There are still those (parents of health cadre) who say that you should not sleep too long, later the baby, when born, will have a lot of white on the skin like fat”. (HCR2)

Midwives elaborated further:

“For naps here (in this community), there are still taboos, parents forbidding pregnant women to taking a nap because they believe that it would make childbirth more difficult”. (MP5)

“In this area, there is still a belief that pregnant women cannot take a nap, even though she is very sleepy. So, the woman is sleep-deprived and tired. it has an effect; the mother's blood pressure measurements are low, and she suffers from anemia too” (MR6)

### 3.2 Theme 2: family hierarchy and power control over pregnant women

Most participants resided in extended households where the pregnant woman and her husband lived with the husband's parents. In such settings, the mother-in-law often exercises decision-making authority regarding the pregnant woman's care, based on her own experiences and traditional knowledge.

“For me, I obey my mother-in-law because she has more experience” (PWP6)

Health cadres reported that pregnant women and their husbands often deferred to the mother-in-law's authority due to socioeconomic constraints and power imbalances:

“My experience tells that when mother-in-law of pregnant woman says this and that, a pregnant woman just answers “yes” because her husband's income is small, she got married at a very younger age, usually husband's mother are the decision makers as she will pay for everything, and determine the place of delivery” (HCP4)

“The parents pay for everything the need of pregnant woman needs, that's why the pregnant women and her husband obeys the parents” (MP6)

Midwives reported that husband's mother is decision maker about health of pregnant woman, some pregnant women and husband still accept their advice:

“In this place (community), the decision maker is husband's mother.” (MP3)

Some women lacked autonomy in managing their reproductive health, including decisions about contraception and family size, resulting in multiple unplanned pregnancies. Deference to the husband's preference for a specific child's gender was also common. One pregnant woman described her experience:

“At first, I used contraceptive pills, but over time I often had dizziness, so I stopped. I wanted to get an implant, but instead I got pregnant. My husband was happy” (PWP6)

Another participant highlighted gender preference as a driver for continued childbearing:

“This is the fourth child. We were expecting a girl because all three siblings were boys. Many people suggested trying once again, we did not have a gender program but when at the ultrasound said the baby was most likely a girl” (HR1)

Some statements from midwives that the husband wanted a certain gender and that pregnant women had become pregnant so that the wishes could be extended to her larger family:

“Before having a son, she already had 9 children but all of them were girls. She is currently pregnant with her 10th child and never had family planning before; her husband did not allow it” (MP2)

“It looks like another boy is coming out, her husband wants a girl, even though this is already gravida 7. I don’t think it will be finished (the pregnancy). She will get pregnant again later” (MR2)

“There was another case, the last delivery (gravida 7) was in 2022, and the reason why she didn’t want family planning was because it wasn’t allowed by her husband, her grandfather, and her mother” (MR4)

### 3.3 Theme 3: cultural practice of shaman during pregnancy

Pregnant women frequently relied on traditional abdominal massage performed by female shamans to reposition the baby and relieve physical discomfort. These massages were believed to alleviate symptoms such as difficulty walking or urinating, which was also believed due to incorrect fetal positioning. The involvement of shamans was often initiated by the mother-in-law:

“If the daughter-in-law was healthy, we did not call the shaman; if she was not feeling well, we called the shaman. Later, when it was 2 or 3 months before giving birth, we called the shaman” (MILP5)

“Sometimes at this stage of pregnancy, pregnant women find it difficult and painful to walk, so we invited the shaman to massage the pregnant woman's abdomen” (MILP6)

However, health cadres expressed concern over the safety of such practices, especially the risk to fetal health:

“In my opinion, I am afraid because there was a fetus in a pregnant woman's abdomen and I am worried that the massage (by shaman) will be harmful to the baby, sometimes if we ask pregnant women, she said that it was ordered by her mother-in-law” (HCB4)

Another health cadre elaborated on the shaman's sustained involvement throughout pregnancy, especially within Javanese traditions:

“If the shaman actually have an active role from the first trimester, she visits pregnant women usually every month to go to her house, massage the abdomen to help position the baby in the abdomen, later the 4-month event is also the shaman who plays an active role, such as Javanese traditions that the shaman directs, there must be events like this and that, then later until the birth usually the shaman accompanies at the public health center” (HCP4)

Midwives recalled past unsafe practices by shamans:

“Old time, when a shaman is assisting a midwife in childbirth, after delivery, she washes her gloves, takes them home and dries them, and uses them for VT (Vaginal toucher), because she wants to be like a midwife” (MB3)

Another midwife reported a fatal consequence linked to shamanic massage:

“There is still a tradition of massaging the abdomen of pregnant women by a shaman, then after that, 2 or 3 days, the baby did not move (died)” (MR2)

## 4 Discussion

This study examined the cultural practices that influenced by power relationship within the pregnant women's family and their potential impact on maternal mortality in the Banjarnegara community. It was observed that many women did not engage in healthy daily routines, were subject to hierarchical family decision-making structures, and relied on traditional shamans whose advice often contradicted medical recommendations—factors that may contribute to increased maternal mortality. This complexity of daily practice of pregnant women power relationship within the family and practice will be presented with cultural and power relationship impact.

### 4.1 Theme 1. Daily activities of pregnant women

Nutritionally, many pregnant women reported avoiding several essential food groups, including protein sources, fruits, vegetables, oils, salt, and sugar. However, balanced nutrition is critical for optimal fetal development. Nutritional needs should be addressed from the earliest stages of life, with pregnancy constituting a vital window for nutritional intervention. Deficiencies during this period can negatively impact on nutritional status of pregnant women such as anemia, underweight, and limited dietary diversity, including pregnancy outcomes (12).

Our findings indicate that poor dietary diversity, imbalanced meals, and short inter-pregnancy intervals were key contributors to anemia. The Indonesian Ministry of Health supports this view, identifying similar factors as primary causes of anemia in pregnancy (13). Lipoeto et al. (14) further reported that chronic energy deficiency in pregnant women increases the risk of anemia (14). Postpartum hemorrhage, the leading cause of maternal mortality, is strongly associated with anemia and grand multiparity (15).

Another cultural practice identified in this study related to rest and sleep patterns. Many pregnant women were discouraged—primarily by husbands and mothers-in-law—from taking daytime naps. However, napping is widely recognized in several cultures, including Mediterranean, Latin American, and Chinese societies, as a beneficial practice. In traditional Chinese culture, for example, afternoon napping is a common and culturally accepted health habit. Research suggests that daytime naps may reduce the risk of low birth weight (16). Conversely, poor sleep quality during pregnancy has been linked to adverse outcomes such as preterm birth, cesarean delivery, maternal pre-eclampsia, and low Apgar scores (17).

### 4.2 Theme 2. Family hierarchy and power control over pregnant women

Family influence—particularly from husbands and mothers-in-law—emerged as a dominant factor shaping women's daily lives in this study. Within household power dynamics, the balance of decision-making between couples was often influenced by the husband's mother, who herself typically married at a young age and lacked adequate knowledge about maternal health. Young age marriage also implies that those couples have less experience of societal and community experience, while their parent-in-laws enable them to support financially. This cultural practice is the norm where young marriage is common within the study area. Young age marriage could impact on decision-making autonomy of the young couples that Cameron et al. (18) study revealed this practice. They reported that in Indonesia, both women and men who married early exhibited reduced involvement in household decision-making. This was attributed to the consequences of child marriage, including limited education, lower-earning job prospects, and reduced household income levels (18). In our study, most of our pregnant women were housewives (unemployed), therefore, they mostly depended on their husbands or mother-in-law for their financial needs including healthcare utilization due to pregnancy. Moreover, one third of our pregnant women had no pregnancy experiences. These might also made the pregnant women more vulnerable to empower themselves to take pregnancy-related decision within the family. The complexity of practice between cultural practice on eating and family power relationship dynamics on decision-making on what to eat is observed in other countries. Another study conducted in South Africa reported that pregnant women chose one or more food practices due to local cultural taboos or beliefs. They were influenced mostly by the taboos and practices of their own mother or grandmother. The common avoided foods were meat products, fish, fruits, beans, eggs, and pumpkin, which were rich in essential micronutrients, protein, and carbohydrates. These foods were avoided for reasons associated with suspected adverse pregnancy outcomes (19).

Under family power dynamics, pregnant women frequently experienced constraints in making decisions about their pregnancy and childbirth. Several participants reported a lack of control over their pregnancies, resulting in limited opportunities to monitor or prioritize their health (20). In some communities, mother/mother-in-law, grandmother/grandmother-in-law or any older female relatives of pregnant women act as a traditional birth attendant and make the decision for delivery. It was observed that when community members sought healthcare services from a formal health system rather than the traditional way, socio-cultural norms significantly influence this choice of place and time for seeking pregnancy, delivery and postnatal care (21). Rizkianti et al. (22) found that women with greater decision-making autonomy during pregnancy were significantly more likely to optimize their health outcomes (22). Observing the current MMR in the study area where the rate indicates the disparities between other area of Indonesia, the micro level of daily practice, in other words, within the family practice that is influenced by power relationship between pregnant women, and their families could be one of the contributing factors.

### 4.3 Theme 3. Cultural practice of shaman during pregnancy

Traditional shamanic practices remained prevalent in the communities studied. Pregnant women commonly sought abdominal massages from shamans to relieve pain, ease movement difficulties, or correct perceived fetal malposition. This practice has been believed by pregnant women and their families to reduce physical discomfort and assist fetal positioning, on the other hand, this practice posed significant risks to both maternal and fetal health. These procedures were conducted solely based on the shaman's experience, with no supporting evidence from scientific research regarding their safety or efficacy. The persistence of such practices was rooted in deeply entrenched cultural beliefs that permeated village life and created resistance to modern, evidence-based healthcare.

As a result, many women continued to give birth at home with the assistance of unskilled traditional birth attendants, increasing the risk of maternal mortality due to complications such as hemorrhage, sepsis, and eclampsia (23). This traditional cultural practice is also observed in various countries where pregnancy and childbirth relied on this approach. For example, Felisian et al. (24) revealed that the traditional practice for Indigenous people in Tanzania (24).

Cultural adherence to traditional health practices and delays in seeking formal medical care significantly contributed to these outcomes (25). The preference for traditional birth attendants (TBAs) was particularly strong in rural and lower income communities (26), as demonstrated by a study in Indonesia, which found that reliance on TBAs was driven by their affordability compared to professional midwives. Due to economic hardship, families often paid TBAs with goods—such as food, livestock, or clothing—in lieu of cash. Furthermore, TBAs were deeply integrated into the cultural fabric of the community and were trusted for generations. They participated in significant cultural rituals throughout the pregnancy and postpartum period, including birth and postnatal ceremonies, which were seen as ancestral legacies (27). To reduce MMR, it is essential to promote safe scientific based practice, however, transforming into medicalized pregnancy care and child birth could easily ignore those daily micro level practice which is strongly embedded into pregnant women and their family living life.

### 4.4 Limitations

This study has several limitations. First, as a qualitative ethnographic investigation, findings are based on participants' self-reported experiences, which may be subject to social desirability bias, particularly on culturally sensitive or stigmatized topics. Second, PHCs were selected conveniently, which might cause selection bias. Moreover, data were collected from only three public health centers, limiting geographic and demographic diversity. As there is ethnic diversity and cultural backgrounds exist in different regions of Indonesia, this may limit the transferability of our study findings. Therefore, considering other local cultures and ethnic groups, these findings require special consideration/results need to be interpreted with caution. Lastly, the PI and research team are familiar with this community, it might also influence data collection and interpretation procedures.

## 5 Conclusion

In conclusion, cultural practices, family dynamics, and traditional health beliefs in Banjarnegara significantly shape the health behaviors of pregnant women. Harmful dietary practices and the dominance of family members in pregnancy-related decisions can negatively impact maternal and fetal outcomes. Continued reliance on shamans and traditional beliefs often leads to unsafe practices, reinforcing maternal health risks. These findings underscore the need for culturally sensitive educational interventions, family awareness regarding healthy power relationships and proper healthcare utilization during pregnancy. Enhanced communication from healthcare providers is essential to dispel harmful myths and encourage the adoption of evidence-based practices. Policymakers should prioritize community awareness initiatives and provide targeted training for healthcare providers to mitigate the detrimental effects of unsafe traditional practices on maternal and neonatal health. For generalizability of the study findings, future research should expand to additional regions and utilize broader, mixed method approaches to gain a more comprehensive understanding of cultural practices affecting maternal health across the country. Moreover, we should emphasize interventional strategies that empower not only pregnant women but also engage family members, especially husbands and mothers-in-law, who hold significant decision-making power.

## Data Availability

The datasets presented in this article are not readily available because of privacy and confidentiality of the participants. Further queries should be directed to dewiesulistyorini@gmail.com.
